# GATA3 induces the pathogenicity of Th17 cells via regulating GM-CSF expression

**DOI:** 10.3389/fimmu.2023.1186580

**Published:** 2023-06-28

**Authors:** Matthew J. Butcher, Rama Krishna Gurram, Xiaoliang Zhu, Xi Chen, Gangqing Hu, Vanja Lazarevic, Keji Zhao, Jinfang Zhu

**Affiliations:** ^1^ Molecular and Cellular Immunoregulation Section, Laboratory of Immune System Biology, National Institute of Allergy and Infectious Diseases, National Institutes of Health, Bethesda, MD, United States; ^2^ Laboratory of Epigenome Biology, Systems Biology Center, National Heart, Lung, and Blood Institute, National Institutes of Health, Bethesda, MD, United States; ^3^ Department of Microbiology, Immunology, and Cell Biology, School of Medicine, West Virginia University, Morgantown, WV, United States; ^4^ Experimental Immunology Branch, National Cancer Institute, National Institutes of Health, Bethesda, MD, United States

**Keywords:** GATA3, experimental autoimmune encephalomyelitis, Th17, pathogenicity, GM-CSF, Bhlhe40

## Abstract

T-bet-expressing Th17 (T-bet^+^RORγt^+^) cells are associated with the induction of pathology during experimental autoimmune encephalomyelitis (EAE) and the encephalitic nature of these Th17 cells can be explained by their ability to produce GM-CSF. However, the upstream regulatory mechanisms that control *Csf2* (gene encoding GM-CSF) expression are still unclear. In this study, we found that Th17 cells dynamically expressed GATA3, the master transcription factor for Th2 cell differentiation, during their differentiation both *in vitro* and *in vivo*. Early deletion of *Gata3* in three complimentary conditional knockout models by Cre-ERT2, *hCd2* ^Cre^ and *Tbx21* ^Cre^, respectively, limited the pathogenicity of Th17 cells during EAE, which was correlated with a defect in generating pathogenic T-bet-expressing Th17 cells. These results indicate that early GATA3-dependent gene regulation is critically required to generate a *de novo* encephalitogenic Th17 response. Furthermore, a late deletion of *Gata3* via Cre-ERT2 in the adoptive transfer EAE model resulted in a cell intrinsic failure to induce EAE symptoms which was correlated with a substantial reduction in GM-CSF production without affecting the generation and/or maintenance of T-bet-expressing Th17 cells. RNA-Seq analysis of *Gata3-*sufficient and *Gata3*-deficient CNS-infiltrating CD4^+^ effector T cells from mixed congenic co-transfer recipient mice revealed an important, cell-intrinsic, function of GATA3 in regulating the expression of *Egr2*, *Bhlhe40*, and *Csf2*. Thus, our data highlights a novel role for GATA3 in promoting and maintaining the pathogenicity of T-bet-expressing Th17 cells in EAE, via putative regulation of Egr2, Bhlhe40, and GM-CSF expression.

## Introduction

As an important part of the adaptive immune system, CD4 T helper (Th) cells play central roles in orchestrating immune responses to a variety of infections as well as during allergic and/or autoimmune reactions via the production of unique sets of cytokines (1). In response to foreign or self-antigen-laden antigen presenting cells, naïve T cells differentiate into distinct Th effector lineages through a combination of T cell receptor (TCR) activation and differentiating cytokine cues. As a result, lineage-specific transcription factors are induced and initiate the differentiation of specific Th effector cell lineages. The master lineage transcription factors for each lineage are T-bet (Th1), GATA3 (Th2), RORγt (Th17), and Foxp3 (Treg), respectively (1, 2). For Th17 cell differentiation, IL-6, IL-21, and/or IL-23 signaling induces the Th17-lineage transcription factor RORγt via Stat3 activation, and RORγt then works in conjunction with the pioneering transcription factors BATF/IRF4 and Stat3 to regulate the expression of effector cytokines IL-17A and IL-17F (1, 3, 4). During Th2 cell differentiation, a combination of TCR stimulation and IL-4-Stat6 signaling is sufficient to drive GATA3 expression and the production of Th2-related cytokines, including IL-4, IL-5, and IL-13 (1). In the case of Th1 cell differentiation, TCR activation together with IL-12- and/or IFNγ-mediated signaling induces T-bet expression and endow T-bet^+^ cells with the capacity to produce IFNγ (1).

However, the one-transcription factor-one fate model is over simplified and there are many *in vivo* experimental contexts in which multiple master lineage transcription factors can be co-expressed (1, 2). In fact, GATA3 is expressed by all T cells at various expression levels *in vivo* and its expression is tightly regulated to an appropriate level for optimal T cell survival and proliferation (1, 5). Additionally, there are multiple instances in which GATA3 is co-expressed at intermediate or high levels with other master-lineage transcription factors. For example, a subset of colonic Foxp3^+^ Tregs can co-express either GATA3 or RORγt, and GATA3/RORγt co-expressing cells have been observed in asthmatic patients and models of allergic inflammation (6, 7). Additionally, in a model of enforced expression of GATA3, *de novo* Th17 cell differentiation was still able to occur, suggesting that GATA3 may not intrinsically block RORγt^+^ Th17 cell differentiation (8). Similarly, T-bet and GATA3 can be co-induced or co-expressed during Th1 differentiation *in vitro* (9–11) and both can be expressed dynamically in Tregs (12). Lastly, there are situations in which T-bet and RORγt can be co-expressed. For example, T-bet^+^RORγt^+^ Th17 cells have been found in the gut and in the central nervous system (CNS), where they are able to co-produce IL-17A and IFNγ (13–17). Thus, while the expression of primary lineage defining transcription factors is critically required for the lineage commitment of Th subsets, dynamic expression of the master regulators of other lineages may endow the established cell lineages with additional functions.

One experimental model in which T-bet^+^RORγt^+^ Th17 cells have garnered significant attention is experimental autoimmune encephalomyelitis (EAE), a mouse model of multiple sclerosis. In EAE, a peripheral immunization with myelin oligodendrocyte glycoprotein peptide (MOG_35-55_) results in the generation of several autoimmune demyelinating Th subsets, including T-bet^–^RORγt^+^ (Th17), T-bet^+^RORγt^+^ (T-bet-expressing Th17), T-bet^+^RORγt^–^ (Th1) cells, which are all found in the CNS at the peak of the disease. Several experimental lines of evidence have shown that 2D2-transgenic *in vitro* polarized Th17 cells are sufficient to induce EAE symptoms in transfer models. However, neither Th1-related IFNγ, nor Th17-related IL-17A, IL-17F, IL-21, IL-22 cytokines are required to provoke EAE symptoms (18–21). Instead, granulocyte-macrophage colony stimulating factor (GM-CSF) has emerged as a key pro-encephalomyelitic cytokine that is both required for EAE and is secreted by encephalitic Th17 and Th1 cells in the CNS (22–24). However, the exact mechanisms through which GM-CSF-production is regulated within T cells are less clear. IL-1β and IL-23 cytokine signaling are required for *in vivo* GM-CSF production during EAE and the transcription factors c-Rel, NF-kB1, RUNX1, RORγt, Bhlhe40 have been proposed to affect T cell *Csf2* expression (22–30). Of the aforementioned transcription factors, *Bhlhe40* is particularly noteworthy as it is induced within T cells upon TCR stimulation and *Bhlhe40^-/-^* mice have been demonstrated to be deficient in GM-CSF production in T cells *in vivo* (31, 32). Thus, Bhlhe40-dependent GM-CSF production within encephalitic T cells has emerged as a key pro-inflammatory pathway in EAE, although the precise mechanisms through which Bhlhe40 expression is regulated are currently unclear.

Innate lymphoid cells (ILCs), the innate counterparts of Th cells, also express T-bet, GATA3 and RORγt, for the development and functions of group 1 ILCs (ILC1s), ILC2s and ILC3s, respectively (33). Like T-bet^+^RORγt^+^ Th17 cells, NKp46^+^ ILC3s in the intestinal lamina propria also express both T-bet and RORγt (34). Strikingly, GATA3 plays important role in the development of NKp46^+^ ILC3s and regulates optimal production of IL-22 (35). Since ILC and Th subsets often utilize similar transcriptional machinery for their development and functions, we hypothesized that GATA3 may also have an important function in regulating the generation and functions of T-bet^+^RORγt^+^ Th17 cells.

Here we report that *de novo* differentiating Th17 cells dynamically express GATA3 ranging from an early intermediate level to a late low level. Complimentary experimental models designed to probe the functions of GATA3 in EAE revealed that while the early intermediate expression of GATA3 is dispensable for the initial differentiation of Th17 cells, it is required to generate encephalitogenic T-bet-expressing Th17 cells and to provoke EAE symptoms. Interestingly, when *Gata3* was deleted at a later stage, following the generation of Th17 cells in the draining lymph node, the re-transfer of these effector T cells in an adoptive transfer EAE model revealed that the production of GM-CSF was drastically reduced without affecting the overall proportion of IFNγ/IL-17A-producing T cells or relative T-bet-expressing Th17 cell percentages. Further co-adoptive transfer experiments revealed that the GATA3-mediated GM-CSF regulation effect was cell intrinsic. Transcriptomic analyses through RNA-Seq revealed that GATA3 regulated the expression of *Bhlhe40* and *Egr2* in a cell-intrinsic manner. Together, these results suggest a novel regulatory pathway involving GATA3, Egr2, Bhlhe40, and GM-CSF in EAE.

## Materials and methods

### Mice

*Gata3* ^fl/fl^ [Taconic line 355 (36)], Cre-ERT2-*Gata3* ^fl/fl^ mice [Taconic line 8445 (37)], *Cd45.1/Cd45.2* C57BL/6 (Taconic line 8422), *Cd45.1* C57BL/6 (Taconic line 7), *Tcra* ^-/-^ (Taconic line 98) and C57BL/6 mice were ordered from the NIAID-Taconic repository or the Taconic. h*Cd2^Cre^Gata3* ^fl/fl^ mice has been reported recently (38). *Tbx21* ^Cre^ mice [Jax line 024507 (39)] were crossed with *Gata3* ^fl/fl^ mice to generate *Tbx21* ^Cre^ *Gata3* ^fl/fl^ mice. *Rorc* ^E2-Crimson^ mice (35) were crossed with *Gata3* ^ZsGreen^ (40) and *Foxp3* ^RFP^ [Jax line 008374 (41)] reporter mice to generate *Rorc* ^E2Crimson^ *Gata3* ^ZsGreen^ *Foxp3* ^RFP^ triple reporter mice. 2D2 mice were purchased from the Jackson Laboratory (JAX line 006912). All mice were imported, bred, and housed within the National Institute of Allergy and Infectious Diseases (NIAID) specific pathogen-free animal facilities. Unless otherwise specified, all experimental mice were used between 6-16 weeks of age under an animal study protocol approved by the NIAID Animal Care and Use Committee.

### *In vitro* CD4 T cell cultures

Naïve T cells (CD3^+^CD4^+^CD45RB^hi^CD25^–^ from C57BL/6 mice or CD3^+^CD4^+^CD45RB^hi^CD25^–^Foxp3^–^RORγt^–^GATA3^–^ from *Gata3* ^ZsGreen^ *Rorc* ^E2-Crimson^ *Foxp3* ^RFP^ mice) were isolated from peripheral lymph nodes via cell sorting (FACSAria, BD Biosciences). The isolated naïve T cells were subsequently cultured under Th17 conditions (1 μg/ml anti-CD3; 2 μg/ml anti-CD28; 10 μg/ml anti-IL-4, 10 μg/ml anti-IFNγ; 0.5 ng/ml TGFβ1, 10 ng/ml IL-1β, 20 ng/ml IL-6, 10 ng/ml IL-23) in complete RPMI1640 media (Invitrogen, 10% FBS (Hyclone), 200 mM Glutamine, 100 mM sodium pyruvate (Gibco), 50 μM β-mercaptoethanol (Sigma), 100 U/ml penicillin and 100 μg/ml streptomycin (Gibco)) for 0-72 hr as indicated.

### Experimental autoimmune encephalomyelitis

For EAE experiments, MOG_35-55_/Complete Freund’s Adjuvant (CFA) and MOG_35-55_/Incomplete Freund’s Adjuvant (IFA) emulsions were prepared. For MOG_35-55_/CFA preparations, 0.4 mg/ml of MOG_35-55_ peptide (MEVGWYRSPFSRVVHLYRNGK, NIAID peptide core facility) was emulsified 1:1 in CFA (BD) supplemented with *Mycobacterium tuberculosis* extract H37Ra (Difco, 4 mg/ml). For MOG_35-55_/IFA preparations, 0.4 mg/ml of MOG35-55 peptide was emulsified 1:1 with IFA (BD).

To induce active EAE, 8-12-week-old sex matched mice were injected subcutaneously with MOG_35-55_/CFA (50μl/flank) and 200 ng of Pertussis Toxin (‘Ptx’, Calbiochem) intraperitoneally on days 0 and 2 of the experiment. Immunized mice were subsequently harvested at the indicated time points or at the peak of EAE symptoms. EAE clinical scores and body weights were collected daily and scored as follows: 0 – asymptomatic, 1 – tail paralysis, 2 – hindlimb paresis, 3 – hindlimb paralysis, 4 – unilateral forelimb paralysis and hindlimb paralysis, 5 – moribund or death. To isolate draining lymph node effector T cells for adoptive cell transfer EAE experiments, the indicated donor mice were immunized with MOG_35-55_/CFA and Pertussis Toxin, and the draining lymph nodes were subsequently collected six days post-immunization. CD3^+^CD4^+^CD44^hi^ T cells were collected from the draining lymph nodes by cell sorting for the cell transfer procedure. For some experiments involving 2D2 cells, naïve 2D2 cells were isolated by cell sorting (CD3^+^CD4^+^CD45RB^hi^CD25^–^Vβ11^+^) and transferred intravenously (2x10^6^ cells/mouse) to *Cd45.1/Cd45.2* hosts before immunization. To induce EAE in *Tcra^-/-^* recipients in adoptive cell transfer EAE experiments, 4x10^6^ donor cells were transferred intravenously and the recipient mice were injected with MOG_35-55_/IFA and Pertussis Toxin (i.p. d0, d2). The *Tcra^-/-^* recipient mice were monitored daily for EAE clinical symptoms as described above. In some experiments involving Cre-ERT2-*Gata3* ^fl/fl^ mice or CD4 T cells, the mice were also injected with 100 μl of tamoxifen (T5648; Sigma-Aldrich, 4mg/ml) or a vehicle control (corn oil) on immunization d0 or cell transfer d0.

### Tissue preparation

For the isolation of CNS-infiltrating cells for flow cytometry experiments, mice were perfused via cardiac puncture with cold PBS immediately following euthanasia. The brain and spinal cord were subsequently dissected, minced finely, and digested with 1 U/ml Liberase TM (05401119001; Roche) and 0.3 U/ml DNase I (10104159001; Roche) in incomplete RPMI1640 media for 30 minutes at 37°C. The tissues were mechanically disrupted via repetitive pipetting and filtered through a 70 μm cell strainer (Fisher Scientific). The resulting cell suspension was centrifuged through a percoll density gradient (38% - 70%) and mononuclear cells were collected from the interphase. The mononuclear cell suspension was washed and resuspended in culture medium for flow cytometry. For the preparation of lymph node or splenic cell suspensions, lymph nodes (inguinal, axillary, brachial) and spleens were isolated sterilely and mechanically disrupted using a 70 μm cell strainer. Erythrocytes were lysed from the resulting splenic cell suspension using ACK lysis buffer (Fisher Scientific). The final cell suspensions were washed and resuspended in culture medium (re-stimulated samples) or FACS buffer (non-stimulated samples) for flow cytometry.

### Flow cytometry and cell sorting

To detect intracellular cytokine production, cells were re-stimulated with 10 ng/ml phorbol 12-myristate 13-acetate (PMA, Sigma Aldrich) and 500 nM ionomycin (Sigma Aldrich) in complete RPMI 1640 media for 5 hours in the presence of 1X Brefeldin A (Biolegend) for the last hour of the incubation. Following re-stimulation, single cell suspensions were first incubated with anti-CD16/CD32 (2.4G2) antibodies (15 minutes, 4°C) and subsequently stained for extracellular antigens (20 minutes, 4°C). Cytokines and transcription factors were stained using the Foxp3 staining buffer set (00-5523-00, eBioscience) according to the manufacturer’s instructions. The samples were acquired using an LSR-II, Fortessa, or FACS Symphony cytometer (BD Biosciences), and the results were analyzed using FlowJo software (Tree Star, v10). The following antibodies were used in the study: CD3 (17A2), CD4 (RM4-5), CD44 (IM7), CD25 (PC61.5), CD45RB (C363-16A), T-bet (04-46), RORγt (Q31-378), GATA3 (TWAJ), Foxp3, IFNγ (XMG1.2), IL-17A (eBio17B7), GM-CSF (MP1-22E9), Bhlhe40 (Dec1, NB100-1800), Egr2 (erongr2), TNFα (MP6-XT22), CD45.1 (A20), CD45.2 (104), Vb11 (RR3-15), CD11b (M1/70), CD45 (30-F11), F4/80 (BM8), Gr1 (RB6-8C5), Tmem119 (106-6), and Goat anti-Rabbit secondary antibody (Thermofisher, A-11008). For FACS sorting experiments, single cell suspensions were prepared sterilely and stained as described above. Specified live cell populations were sorted using a FACS Aria (BD Biosciences) and collected into complete RPMI 1640 media. The isolated populations were washed twice with PBS and subsequently used for downstream applications.

### RNA-Seq analysis

CNS-infiltrating CD3^+^CD4^+^CD44^hi^CD45.1^+^CD45.2^+^ and CD3^+^CD4^+^CD44^hi^CD45.1^–^CD45.2^+^ T cells were sorted directly into 300 μl of Qiazol (Qiagen) from vehicle or tamoxifen treated *Cd45.1/Cd45.2* C57BL/6 and *Cd45.2* Cre-ERT2-*Gata3* ^fl/fl^ mixed co-transfer EAE *Tcra^-/-^* hosts by cell sorting. Total RNA was extracted and cDNA libraries were prepared using the Smart-Seq2 method (42) as previously described (43). Multiplex sequencing reads of 50 bp were generated by the NHLBI DNA Sequencing and Computational Biology Core and sequence reads were mapped to the mouse genome (mm9) using bowtie 2 with the default settings (44). Gene expression was measured by RPKM (45) and differentially expressed genes were identified using Partek Flow (Partek). Differentially expressed genes were imported into Ingenuity Pathway Analysis and analyzed using the Core Analysis settings. Th17-related genes and pathways that connect *Gata3*, *Egr2*, *Bhlhe40*, and *Csf2* were built using the differential expression data, and the build and connect features of Ingenuity Pathway Analysis. The RNA-Seq datasets have been deposited at the Gene Expression Omnibus database under the accession no. GSE227394.

### Statistics

Statistical differences between experimental groups were determined by a two-tailed Student’s *t* test, Bonferroni-holm multiple comparison-corrected Student’s *t* tests, or one way ANOVA with Tukey post-hoc comparison tests as appropriate with Prism 7 software. For all statistical comparisons, *, p <0.05; **, p <0.01; ***, p <0.001. All summary data are reported as mean ± standard error of the mean.

## Results

### GATA3 is dynamically expressed during *de novo* Th17 cell differentiation both *in vitro* and *in vivo*

**Figure 1:**
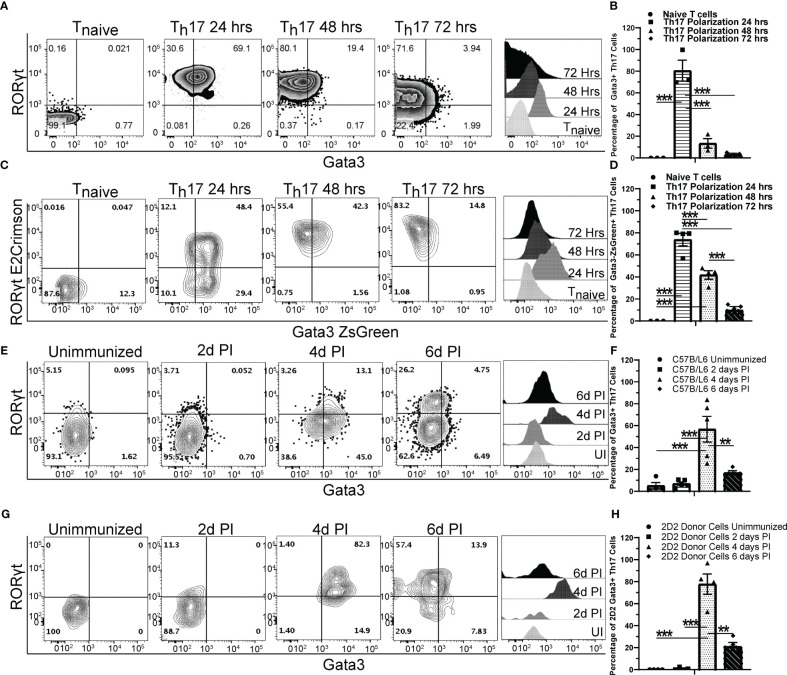
GATA3 is transiently induced during Th17 cell differentiation. **(A, B)** Naïve C57BL/6 CD4 T cells cultured under Th17 polarization conditions and monitored for GATA3 expression at 0, 24, 48, and 72 hr. **(A)** Representative FACS plots at the indicated timepoints depicting GATA3 and RORγt staining amongst CD4^+^CD44^+^Foxp3^-^ T cells and representative GATA3 histograms within CD4^+^CD44^+^Foxp3^-^RORγt^+^ T cells. **(B)** Mean % of GATA3-expressing (i.e., GATA3^+^RORγt^+^ Th17) cells among CD4^+^CD44^+^Foxp3^-^RORγt^+^ populations in **(A)** from three independent experiments. **(C, D)** Representative RORγt and GATA3 reporter expression by *Rorc* ^E2-Crimson^ *Gata3* ^ZsGreen^ *Foxp3* ^RFP^ CD4 T cells cultured under Th17 polarization conditions as in **(A)**. **(D)** Mean % of GATA3-expressing (i.e., GATA3^+^RORγt^+^ Th17) cells among CD4^+^CD44^+^Foxp3^-^RORγt^+^ populations in **(C)** from two independent experiments. **(E–H)** The *in vivo* kinetics of GATA3 expression within draining lymph node (dLN) Th17 cells in response to MOG_35-55_/CFA immunization. **(E, F)** Representative GATA3 and RORγt staining **(E)** within CD4^+^CD44^+^Foxp3^-^ dLN T cells and the mean % of GATA3-expressing cells among CD4^+^CD44^+^Foxp3^-^RORγt^+^ T cells **(F)** from immunized C57BL/6 mice (n=5-8 mice/timepoint from three independent experiments). **(G, H)** Representative GATA3 and RORγt staining **(G)** within donor 2D2 CD4^+^CD44^+^Foxp3^-^ dLN T cells and the mean % of GATA3-expressing cells among 2D2 CD4^+^CD44^+^Foxp3^-^RORγt^+^ T cells **(H)** from immunized 2D2 naïve T cell recipient *Cd45.1/Cd45.2* mice (n=4 mice/timepoint from two independent experiments). UI – unimmunized, PI – post immunization. For statistical comparisons, a one-way ANOVA was conducted with Tukey *Post-Hoc* testing for group comparisons. Significance levels are denoted as follows: **p <0.01; ***p <0.001.

GATA3 is important for the development of NKp46^+^ ILC3s that express both T-bet and RORγt (35). To test whether GATA3 also plays a role in T-bet-expressing Th17 cells, we first examined the kinetics of GATA3 expression by flow cytometry during *de novo* Th17 polarization *in vitro* (**Figures 1A, B**). As expected, naïve T cells expressed a low baseline level of GATA3. However, the expression of both GATA3 and RORγt was induced within 24 hours of culture. GATA3 expression was then gradually reduced to lower levels over the next 48 hours of culture. The dynamic expression of GATA3 in RORγt-expressing cells was further assessed using naïve T cells from the *Gata3* ^ZsGreen^ *Rorc* ^E2-Crimson^ *Foxp3* ^RFP^ triple reporter mice (**Figures 1C, D**). Again, GATA3-ZsGreen and RORγt-E2-Crimson were co-induced within 24 hours of Th17-polarizing culture conditions, and GATA3-ZsGreen expression was subsequently reduced back to a low level over the next 48 hours. We next examined the kinetics of GATA3 expression in differentiating Th17 cells *in vivo* using MOG_35-55_/CFA immunized C57BL/6 mice (**Figures 1E, F**). In the unimmunized naïve C57BL/6 mice, few RORγt^+^ Th17 cells were present within the lymph nodes and all of them were GATA3^low^. On the fourth day post immunization, CD4 T cells in the draining lymph nodes began to co-express GATA3 and RORγt, however, by the sixth day post immunization, GATA3 expression within the RORγt^+^ Th17 cells returned to a low state akin to naïve T cells. We also assessed MOG-antigen specific CD45.2 + 2D2 cells that were adoptively transferred to CD45.1^+^CD45.2^+^ host mice which were subsequently immunized with MOG_35-55_/CFA (**Figures 1G, H**). As expected, in the unimmunized state, CD45.2^+^ naïve 2D2 T cells retained a naïve phenotype and did not express RORγt or GATA3 within the naïve CD45.1^+^CD45.2^+^ hosts. However, the donor 2D2 T cells co-expressed GATA3 and RORγt on the fourth day post-immunization and the RORγt^+^ Th17 cells downregulated GATA3 to a low state on the sixth day post-immunization. Taken together, these data demonstrate that GATA3 is dynamically regulated during a *de novo* Th17 cell differentiation both *in vitro* and *in vivo*.

### Early expression of GATA3 is essential to generate pathogenic T-bet^+^ Th17 cells and to induce EAE

**Figure 2:**
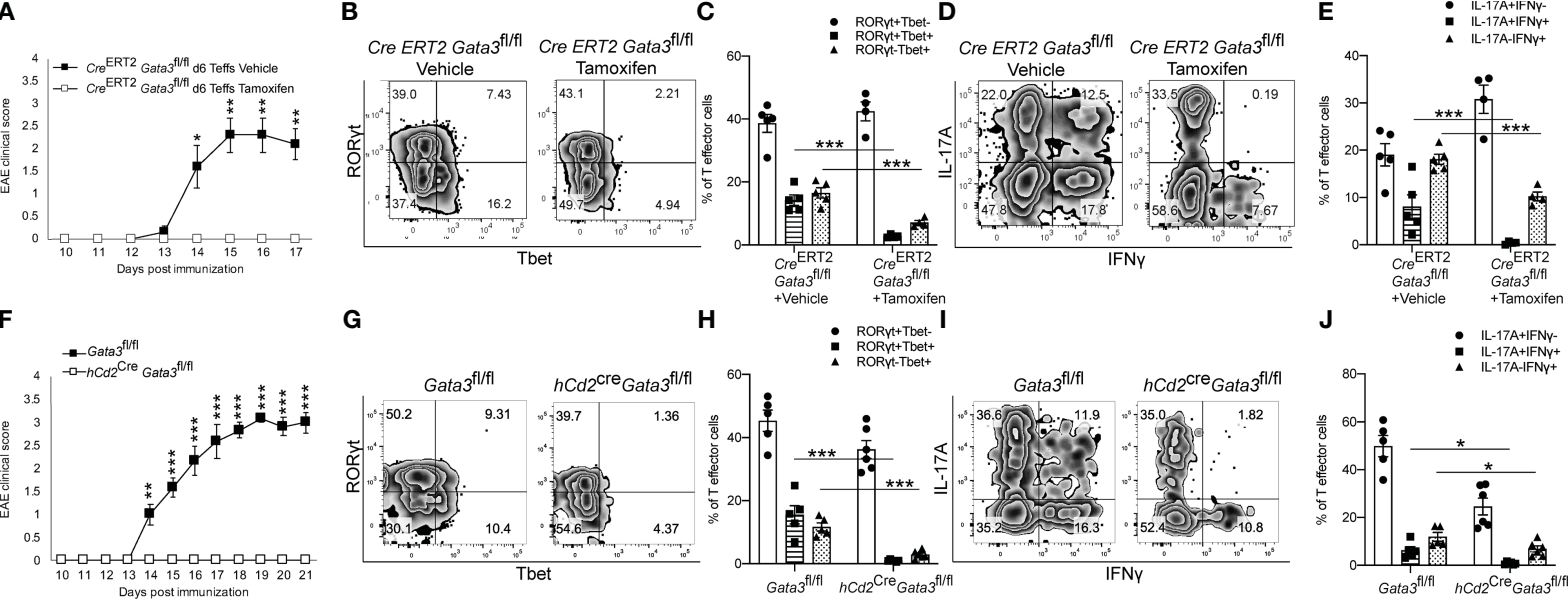
Early GATA3 expression is required to generate a *de novo* T-bet^+^ Th17 cell response and EAE symptoms. **(A)** Mean EAE clinical scores from *Tcra^-/-^* hosts that received 4x10^6^ CD4^+^CD44^+^ T cells harvested from the draining lymph nodes (dLNs) of Cre-ERT2-*Gata3* ^fl/fl^ mice 6 days after MOG/CFA immunization with vehicle or tamoxifen treatment (n=12 mice/group from three independent experiments). On the day of the cell transfer procedure, the *Tcra^-/-^* host mice were boosted with MOG_35-55_/IFA and Pertussis Toxin as described in the methods. **(B, C)** Representative T-bet and RORγt staining amongst d0 Vehicle or Tamoxifen-treated Cre-ERT2-*Gata3* ^fl/fl^ d6 dLN CD3^+^CD4^+^CD44^+^Foxp3^-^ T cells **(B)**, and the mean percentages of T-bet and RORγt expressing T cell subsets **(C)**. **(D, E)** Representative IFNγ and IL-17A staining amongst Vehicle or Tamoxifen treated Cre-ERT2-*Gata3* ^fl/fl^ d6 dLN CD3^+^CD4^+^CD44^+^Foxp3^-^ T cells **(D)**, and the mean percentages of IFNγ and IL-17A expressing T cell subsets (**E**, n =5 mice/group from two independent experiments). **(F)** Mean EAE clinical scores from MOG_35-55_/CFA immunized *Gata3* ^fl/fl^ and *hCd2* ^Cre^ *Gata3* ^fl/fl^ mice. n=12 mice/group from three independent experiments. **(G, H)** Representative T-bet and RORγt staining amongst *Gata3* ^fl/fl^ and *hCd2* ^Cre^ *Gata3* ^fl/fl^ d6 post-immunization dLN CD3^+^CD4^+^CD44^+^Foxp3^-^ T cells **(G)**, and the mean percentages of T-bet and RORγt expressing T cell subsets **(H)**. **(I, J)** Representative IFNγ and IL-17A staining amongst *Gata3* ^fl/fl^ and *hCd2* ^Cre^ *Gata3* ^fl/fl^ d6 dLN CD3^+^CD4^+^CD44^+^Foxp3^-^ T cells **(I)**, and the mean percentages of IFNγ and IL-17A expressing T cell subsets **(J)**. n=6 mice/group from two independent experiments. For statistical comparisons, unpaired student’s T tests were used. Significance levels are denoted as follows: *p <0.05; **p <0.01; ***p <0.001.

To determine what effects early GATA3 expression might have on the development of a Th17 cell response *in vivo*, we utilized three complimentary *Gata3* conditional knockout mouse strains in the EAE model. First, we immunized the Cre-ERT2-*Gata3* ^fl/fl^ mice with MOG/CFA with or without tamoxifen pretreatment on day 0. CD4 T cells from the draining lymph nodes (dLNs) of these immunized mice were isolated on day 6 post immunization and then transferred into the *Tcra* ^-/-^ recipient mice. In this adoptive transfer EAE experiments, CD4 T effector cells from tamoxifen-pretreated Cre-ERT2-*Gata3* ^fl/fl^ dLNs were unable to elicit EAE symptoms in new *Tcra* ^-/-^ hosts, in comparison to CD4 effector cells from vehicle-treated Cre-ERT2-*Gata3* ^fl/fl^ dLNs (**Figure 2A**). To assess the effects of deleting *Gata3* on the development of ‘non-pathogenic’ or ‘pathogenic’ Th17 cells in MOG-immunized mice, we quantified the frequency of Th subsets that either expressed T-bet and RORγt (**Figures 2B, C**) or IFNγ and IL-17A (**Figures 2D, E**) in dLNs six days post immunization. Interestingly, tamoxifen pre-treated Cre-ERT2-*Gata3* ^fl/fl^ mice failed to generate a ‘pathogenic’ T-bet^+^RORγt^+^ Th17 cell response in comparison to vehicle-treated controls (**Figures 2B, C**); which corresponded with a failure to generate IFNγ^+^IL-17A^+^ Th17 cells (**Figures 2D, E**). However, since many other cell types, including ILCs, NK cells, NKT, and CD8 T cells, rely on GATA3 for their development and functionality, and recent publications have suggested that meningeal NKp46^+^ ILCs help to regulate Th17 cell-mediated neuroinflammation in the CNS (46, 47), we were concerned that the failure to mount a T-bet^+^RORγt^+^ Th17 response might be reflective of the functions of GATA3 in ILCs rather than a T cell intrinsic defect. To rule out the functions of GATA3 in non-T cells, we crossed *hCd2* ^Cre^ mice with *Gata3* ^fl/fl^ mice to assess the effects of a complimentary mature T cell-restricted *Gata3* knockout (*hCd2* ^Cre^ *Gata3* ^fl/fl^) on EAE induction (**Figures 2F, J**). Interestingly, *hCd2* ^Cre^ *Gata3* ^fl/fl^ mice similarly failed to develop EAE in comparison to *Gata3* ^fl/fl^ controls (**Figure 2F**). In addition, *hCD2* ^Cre^Gata3^fl/fl^ mice failed to generate a T-bet^+^RORγt^+^ Th17 cell response within the draining lymph nodes in comparison to *Gata3* ^fl/fl^ mice (**Figures 2G, H**); which also corresponded with a failure to generate IFNγ^+^IL-17A^+^ Th17 cells (**Figures 2I, J**). These data demonstrate that early GATA3 expression during *de novo* Th17 cell differentiation is required to generate a pathogenic T-bet^+^RORγt^+^ Th17 cell response.

**Figure 3:**
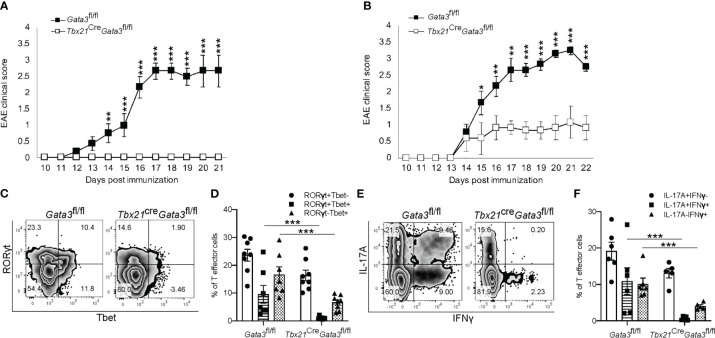
GATA3 expression is required for the development and/or maintenance of early T-bet^+^ Th17 cells. **(A)** Mean EAE clinical scores from MOG_35-55_/CFA immunized *Gata3* ^fl/fl^ and *Tbx21* ^Cre^ *Gata3* ^fl/fl^ mice. n=10 mice/group from three independent experiments. **(B)** Mean cell transfer EAE clinical scores from *Tcra^-/-^* mice that received d6 dLN *Gata3* ^fl/fl^ or *Tbx21* ^Cre^ *Gata3* ^fl/fl^ donor CD4^+^CD44^hi^ T cells and immunized with MOG_35-55_/IFA. n=10 mice/group from three independent experiments. **(C, D)** Representative T-bet and RORγt staining amongst *Gata3* ^fl/fl^ and *Tbx21* ^Cre^ *Gata3* ^fl/fl^ d6 post-immunization dLN CD3^+^CD4^+^CD44^hi^Foxp3^-^ T cells **(C)**. The mean percentages of T-bet- and RORγt-expressing CD4 T cell subsets within the d6 dLN **(D)**. **(E, F)** Representative IFNγ and IL-17A staining amongst *Gata3* ^fl/fl^ and *Tbx21* ^Cre^ *Gata3* ^fl/fl^ d6 post-immunization dLN CD3^+^CD4^+^CD44^hi^Foxp3^-^ T cells **(E)**. The mean percentages of IFNγ and IL-17A positive dLN CD3^+^CD4^+^CD44^hi^Foxp3^-^ T cell subsets. n=6 mice/group from two independent experiments. For statistical comparisons, unpaired student’s T tests were used. Significance levels are denoted as follows: *p <0.05; **p <0.01; ***p <0.001.

Next, to determine whether GATA3 is required in pathogenic T-bet^+^RORγt^+^ Th17 cells, we crossed *Tbx21* ^Cre^ mice with *Gata3* ^fl/fl^ mice to generate a *Gata3* conditional knockout mouse model with GATA3 deficiency only in T-bet-expressing/expressed cells (*Tbx21* ^Cre^ *Gata3* ^fl/fl^). Again, *Tbx21* ^Cre^ *Gata3* ^fl/fl^ mice were resistant to developing EAE symptoms in comparison to *Gata3* ^fl/fl^ controls (**Figure 3A**). However, as T-bet-expressing NKp46^+^ meningeal ILCs (46, 47) have been reported to play a critical role in regulating Th17-mediated neuroinflammation and GATA3 regulates the development and functionality of ILC1 and NK cells, we sought to test the role of GATA3 in T-bet^+^RORγt^+^ Th17 cells through the adoptive transfer model of EAE (**Figure 3B**). In transfer EAE experiments, CD4 effector cells harvested from dLNs of the *Tbx21* ^Cre^ *Gata3* ^fl/fl^ 6 days post immunization were only able to elicit mild EAE symptoms in comparison to *Gata3* ^fl/fl^ CD4 effector cells (**Figure 3B**), suggesting that the resistance to EAE conferred by the *Tbx21* ^Cre^ *Gata3* ^fl/fl^ conditional knockout is mediated by T-bet^+^ T cells. *Tbx21* ^Cre^ *Gata3* ^fl/fl^ mice were unable to generate and/or maintain T-bet^+^RORγt^+^ Th17 cells within the draining lymph nodes of immunized mice (**Figures 3C, D**). In addition, as we observed before, *Tbx21* ^Cre^ *Gata3* ^fl/fl^ CD4 effector cells were also unable to generate IFNγ^+^IL-17A^+^ Th17 cells (**Figures 3E, F**). Together, all our results demonstrate that T-bet^+^ Th17 cells require early GATA3 expression during *de novo* Th17 cell differentiation for their development and encephalitic functions in EAE.

### GATA3 is essential for GM-CSF expression by pathogenic T-bet^+^RORγt^+^ Th17 cells

**Figure 4:**
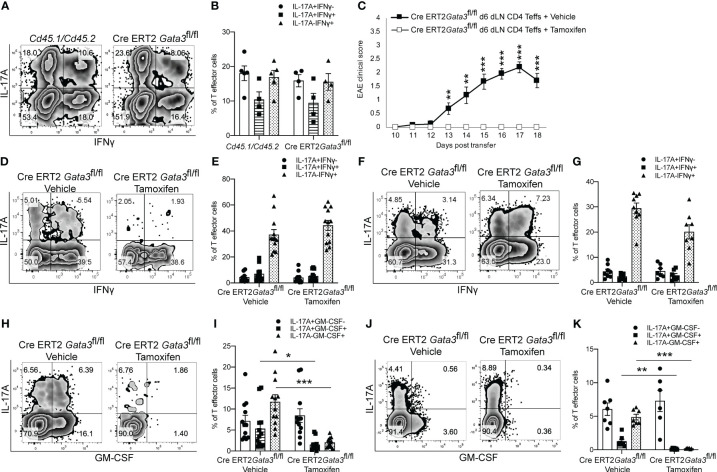
GATA3 is required for GM-CSF production and CD4 T cell-mediated encephalomyelitis. **(A, B)** Characterization of the pre-transfer d6 dLN CD3^+^CD4^+^CD44^hi^ *Cd45.1/Cd45.2* and Cre-ERT2-*Gata3* ^fl/fl^ populations. **(A)** Representative IFNγ vs IL-17A staining and **(B)** the mean percentages of IFNγ and IL-17A CD4^+^CD44^hi^Foxp3^-^ T cell subpopulations in the pre-transfer isolates. **(C)** Mean transfer EAE clinical scores from *Tcra^-/-^* hosts that received 4x10^6^ Cre-ERT2-*Gata3* ^fl/fl^ d6 dLN CD4^+^CD44^hi^ T cells **(A, B)** and a vehicle or tamoxifen treatment. n=15 mice/group from four independent experiments. (D-G; H-K) Characterizations of the Cre-ERT2-*Gata3* ^fl/fl^ donor CD4 T cells within vehicle or tamoxifen treated *Tcra^-/-^* hosts post-transfer. Representative IFNγ and IL-17A staining of vehicle or tamoxifen treated Cre-ERT2-*Gata3* ^fl/fl^ donor CD4 T cells in the *Tcra* ^-/-^ host CNS **(D)** and the spleen **(F)**. The mean percentages of IFNγ and IL-17A subpopulations within the CNS **(E)** and spleen **(G)**. Representative GM-CSF and IL-17A staining of vehicle or tamoxifen treated Cre-ERT2-*Gata3* ^fl/fl^ donor CD4 T cells within the *Tcra* ^-/-^ host CNS **(H)** and the spleen **(J)**. The mean percentages of IL-17A and GM-CSF positive donor CD4 T effector cells within the CNS **(I)** and the spleen **(K)**. n=12 recipient mice/group from four independent experiments. For statistical comparisons, unpaired student’s T tests were used. Significance levels are denoted as follows: *p <0.05; **p <0.01; ***p <0.001.

**Figure 5:**
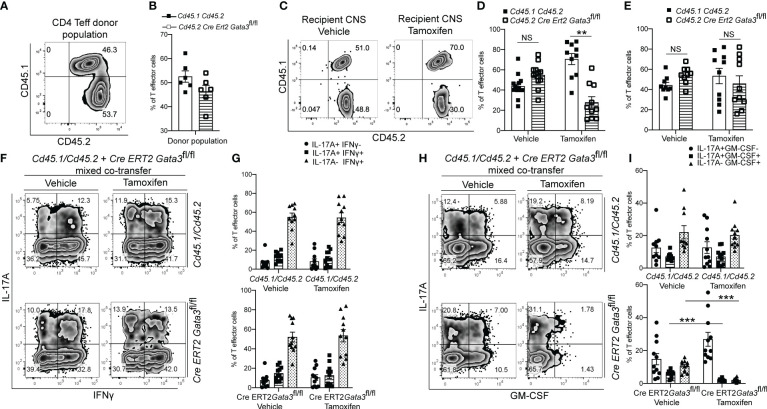
The post-differentiation effects of GATA3 on GM-CSF production is cell intrinsic. Mixed co-transfers of CD45.1^+^CD45.2^+^ C57BL/6 and CD45.2^+^ Cre-ERT2-*Gata3* ^fl/fl^ d6 dLN CD4^+^ T effector cells were conducted. **(A, B)** The starting ratios of FACS-sorted donor *Cd45.1/Cd45.2* C57BL/6 and *Cd45.2* Cre-ERT2-*Gata3* ^fl/fl^ d6 dLN CD4 T effector cells shown as a representative FACS plot **(A)** and population means **(B)**. **(C-E)** The post-transfer ratios of donor CD45.1^+^CD45.2^+^ C57BL/6 and CD45.2^+^ Cre-ERT2-*Gata3* ^fl/fl^ cells within host *Tcra* ^-/-^ mice treated with corn oil (vehicle) or tamoxifen. **(C)** Representative CD45.2 and CD45.1 staining within the CNS. The mean percentages of corn oil or tamoxifen treated CD45.1^+^CD45.2^+^ C57BL/6 and CD45.2^+^ Cre-ERT2-*Gata3* ^fl/fl^ CD4 T cells within the CNS **(D)** and spleen **(E)**. **(F, G)** Representative post-transfer corn oil or tamoxifen treated CD45.1/CD45.2 and Cre-ERT2-*Gata3* ^fl/fl^ donor CD4 T effector IFNγ and IL-17A staining **(F)** and subpopulation means **(G)** within the CNS of *Tcra* ^-/-^ recipient mice. **(H, I)** Representative post-transfer donor *Cd45.1/Cd45.2* and Cre-ERT2-*Gata3* ^fl/fl^ donor CD4 T effector GM-CSF and IL-17A staining **(H)** and subpopulation means **(I)** within the CNS of *Tcra* ^-/-^ recipient mice. n=12 mice/group from three independent experiments. For statistical comparisons, unpaired student’s T tests were used. Significance levels are denoted as follows: *p <0.05; **p <0.01; ***p <0.001.

As we found that GATA3 is induced and subsequently downregulated to a low expression state during Th17 cell differentiation *in vivo*, and that early GATA3 expression is essential for the generation of T-bet^+^RORγt^+^ Th17 cells, we were curious as to what effects late post-differentiation maintenance levels of GATA3 might have on the pathogenicity of established Th17 cells in EAE. To examine how late maintenance levels of GATA3 might affect the pathogenicity of Th17 cells in EAE, we assessed the effects of a late *Gata3* deletion on the pathogenicity of Cre-ERT2-*Gata3* ^fl/fl^ CD4 effector cells in transfer EAE experiments. We first compared the frequency of IFNγ and IL-17A positive CD4 effector cells within the d6 draining lymph nodes of *Cd45.1/Cd45.2* and Cre-ERT2-*Gata3* ^fl/fl^ mice (**Figures 4A, B**). As expected, the frequencies of IFNγ and IL-17A positive CD4 effector cells were similar amongst *Gata3*-sufficient *Cd45.1/Cd45.2* and Cre-ERT2-*Gata3* ^fl/fl^ mice, confirming that IFNγ^+^IL-17A^+^ Th17 cells were efficiently generated before deletion of *Gata3*. To assess the effects of a late *Gata3* deletion on the pathogenicity of Cre-ERT2-*Gata3* ^fl/fl^ effector cells, d6 dLN CD4 effector cells were transferred to *Tcra* ^-/-^ recipients, which were subsequently treated with corn oil (vehicle) or Tamoxifen on post transfer day 0 (**Figure 4C**). Interestingly, Tamoxifen-treated Cre-ERT2-*Gata3* ^fl/fl^ CD4 effector cells *Tcra^-/-^* recipients were resistant to transfer EAE in comparison to vehicle treated controls (**Figure 4C**), indicating that late maintenance levels of GATA3 are also required for Th17-mediated encephalomyelitis. To determine how a late *Gata3* deletion might affect the frequency of IFNγ- and IL-17A-producing CD4 effector cells, we phenotyped donor *Gata3*-sufficient and deficient effector cells from transfer EAE recipient mice. In contrast to the early *Gata3* deletion model in which *Gata3*-deficient cells were unable to generate T-bet^+^RORγt^+^ Th17 cells, deleting *Gata3* at the post-differentiation stage did not affect the frequency of IFNγ or IL-17A positive CD4 effector cells in the CNS (**Figures 4D, E**) nor in the spleen (**Figures 4F, G**). Instead, a late *Gata3*-deletion resulted in a substantial reduction in GM-CSF-producing Cre-ERT2-*Gata3* ^fl/fl^ donor CD4 effector cells in the CNS (**Figures 4H, I**) and the spleen (**Figures 4J, K**). As prior work has demonstrated that GM-CSF is an effector cytokine critically required for the recruitment and activation of CNS mononuclear cells and for EAE induction (22, 23, 48), the inability of late tamoxifen-treated Cre-ERT2-*Gata3* ^fl/fl^ CD4 effector cells to produce GM-CSF likely explains why these transferred CD4 effector cells were unable to induce EAE. However, as the cytokines IL-23 and IL-1β are required for GM-CSF induction (48), we were concerned that the effects of late GATA3^low^ expression on GM-CSF production might be reflective of a less inflammatory environment rather than a cell intrinsic effect. Thus, to determine if a late deletion of *Gata3* affects GM-CSF production in a cell intrinsic manner, we conducted mixed congenic co-transfer EAE experiments. In brief, *Cd45.1/Cd45.2* and *Cd45.2* Cre-ERT2-*Gata3* ^fl/fl^ d6 dLN CD4^+^ T effector cells were collected from MOG_35-55_/CFA immunized donor mice, mixed at a 1:1 ratio, and then transferred to vehicle- or tamoxifen-treated *Tcra* ^-/-^ recipients. We first assessed the relative percentages of *Cd45.1/Cd45.2* and Cre-ERT2-*Gata3* ^fl/fl^ donor cells pre-transfer (**Figures 5A, B**) and post-transfer in the vehicle- or tamoxifen-treated *Tcra* ^-/-^ recipients’ CNS (**Figures 5C, D**) and spleen (**Figure 5E**). *Cd45.1/Cd45.2* and Cre-ERT2-*Gata3* ^fl/fl^ donor CD4 effector cells were equally present in the starting population (**Figures 5A, B**) and in the vehicle- and tamoxifen-treated *Tcra* ^-/-^ recipient spleens following the transfer (**Figure 5E**). However, we detected a slight reduction in the frequency of CNS-infiltrating Cre-ERT2-*Gata3* ^fl/fl^ effector cells within the tamoxifen-treated *Tcra* ^-/-^ mice (**Figures 5C, D**) *vs* vehicle control recipients, suggesting that a late *Gata3* deletion confers a slight cell-intrinsic disadvantage to *Gata3*-deficient Cre-ERT2-*Gata3* ^fl/fl^ effector cells in comparison to *Gata3*-sufficient effector cells (**Figures 5C, D**). We next assessed the phenotypes of the donor cells in the CNS (**Figures 5F–I**). As in our single population transfer EAE experiments (**Figure 4**), we observed a similar distribution of IFNγ and IL-17A positive *Cd45.1/Cd45.2* and Cre-ERT2-*Gata3* ^fl/fl^ donor CD4 effector cells in the vehicle-treated and tamoxifen-treated *Tcra* ^-/-^ recipients. However, when we assessed *Cd45.1/Cd45.2* and Cre-ERT2-*Gata3* ^fl/fl^ CD4 T effector cells for their ability to produce GM-CSF, we noticed that GM-CSF staining was dramatically reduced in a cell-intrinsic manner in the tamoxifen-induced *Gata3*-knockout Cre-ERT2-*Gata3* ^fl/fl^ effector cells (**Figures 5H, I**). Thus, these data together suggest that a late *Gata3* deletion does not affect the maintenance, stability, or ability of Th17 cells to generate T-bet^+^ Th17 cells; instead, encephalitic CD4 T cells intrinsically require low levels of GATA3 to efficiently produce GM-CSF.

### GATA3 is required for normal expression of *Bhlhe40*, *Egr2* and *Csf2*

**Figure 6:**
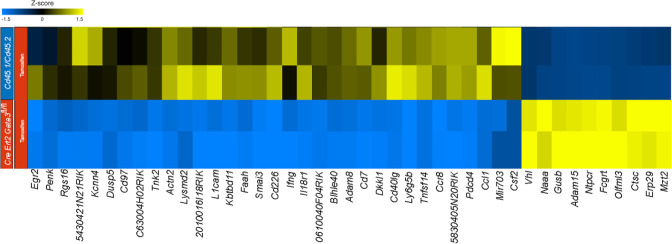
GATA3 regulates pro-inflammatory gene expression in EAE. RNA-Seq analysis was performed using CNS-infiltrating CD4^+^CD44^hi^CD25^-^CD45.1^+^CD45.2^+^ C57BL/6 and CD45.1^-^CD45.2^+^ Cre-ERT2-*Gata3* ^fl/fl^ donor T effector cells from tamoxifen-treated *Tcra^-/-^* co-transfer EAE mice at the peak. Differentially expressed genes were identified using Partek Flow Genomic Suite (Partek) and curated based on an FDR threshold of <0.05. Differentially expressed genes were clustered and displayed as a heatmap. The results are representative of biological duplicates.

To gain insight as to how late expression of GATA3 might regulate GM-CSF within established CD4 effector cells, we compared the transcriptomes of CNS-infiltrating *Gata3*-sufficient and *Gata3*-deficient CD4 effector cells in the mixed *Cd45.1/Cd45.2* and Cre-ERT2-*Gata3* ^fl/fl^ co-transfer EAE model (**Figure 6**) at the peak of EAE. Comparison of gene expression between CNS-infiltrating *Cd45.1/Cd45.2* and Cre-ERT2-*Gata3* ^fl/fl^ CD4^+^CD44^hi^ T effector cells that were isolated from the same vehicle-treated *Tcra* ^-/-^ recipients did not reveal much differentially regulated genes. On the other hand, by comparing the transcriptomes of CNS-infiltrating *Cd45.1/Cd45.2* and Cre-ERT2-*Gata3* ^fl/fl^ CD4^+^CD44^hi^ T effector cells isolated from tamoxifen-treated *Tcra* ^-/-^ recipients at the peak of EAE, we identified 97 differentially expressed genes, of which 72 were significantly down-regulated in *Gata3*-deficient effector cells. Genes associated with Th1-related responses including *Ifng, Il2, Penk, Ccl1*, and *Il18r1*, genes associated with T cell-B cell signaling including *Cd40lg*, *Tnfsf11*, and *Tnfsf14*, and *Csf2* expression, consistent with our results above (**Figures 4**, **5**), were downregulated (**Figure 6**). Interestingly, we also detected altered expression of several transcription regulators, including down-regulated expression of *Bhlhe40* and *Egr2*, and up-regulated expression of *Vhl* in *Gata3*-deficient Cre-ERT2-*Gata3* ^fl/fl^ vs *Gata3*-sufficient *Cd45.1/Cd45.2* T effector cells. *Vhl*, *Egr2*, and *Bhlhe40* are of note as *Vhl* is an important regulator of the HIF1a hypoxic-response pathway in T cells (49) and *Vhl* has been implicated as a potential upstream regulator of *Bhlhe40* (*Stra13*) in human RCC4 cells (50). In addition, Miao and colleagues have shown that Egr2 can bind to the *Bhlhe40* locus within CD4 T cells in ChIP experiments (51), and *Bhlhe40* has been shown to directly regulate *Csf2* expression in knockout and ChIP experiments (31, 52).

**Figure 7:**
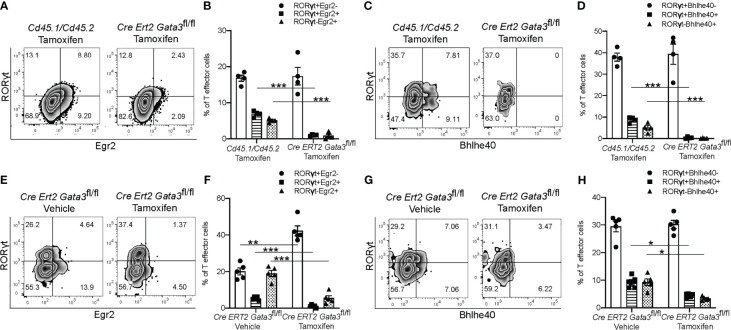
GATA3 is required for normal Bhlhe40 and Egr2 expression at all stages. **(A–D)** The expression of Egr2 and Bhlhe40 within *Gata3*-sufficient (*Cd45.1/Cd45.2*) and *Gata3*-deficient (Cre-ERT2-*Gata3* ^fl/fl^) CNS-infiltrating CD3^+^CD4^+^CD44^hi^Foxp3^-^ T effector cells from co-transfer EAE *Tcra^-/-^* recipient mice treated with tamoxifen (cell transfer d0). **(A)** Representative Egr2 and RORγt staining and summary statistics **(B)** amongst tamoxifen treated CNS-infiltrating CD4^+^CD44^hi^Foxp3^-^ *Cd45.1/Cd45.2* and Cre-ERT2-*Gata3* ^fl/fl^ cells. **(C)** Representative Bhlhe40 and RORγt staining and summary statistics **(D)** amongst tamoxifen treated CD4^+^CD44^hi^Foxp3^-^ *Cd45.1/Cd45.2* and Cre-ERT2-*Gata3* ^fl/fl^ cells. n=4 mice/condition from two independent experiments. **(E–H)** The expression of Egr2 and Bhlhe40 within *Gata3*-sufficient (Vehicle, d0) or *Gata3*-deficient (Tamoxifen, d0) day 6 dLN CD3^+^CD4^+^CD44^hi^Foxp3^-^ T effector cells from MOG_35-55_/CFA-immunized Cre-ERT2-*Gata3* ^fl/fl^ mice. **(E)** Representative Egr2 and RORγt staining and the corresponding summary statistics **(F)** from vehicle control or tamoxifen treated d6 dLN Cre-ERT2-*Gata3* ^fl/fl^ CD4 T effector cells. **(G)** Representative d6 dLN CD4 T effector Bhlhe40 and RORγt staining and the corresponding summary statistics **(H)** from immunized and vehicle or tamoxifen treated Cre-ERT2-*Gata3* ^fl/fl^ mice. n=4 mice/condition from two independent experiments. For statistical comparisons, unpaired student’s T tests were used. Significance levels are denoted as follows: *p <0.05; **p <0.01; ***p <0.001.

We further confirmed the regulation of *Egr2 and Bhlhe40* expression by GATA3 at the protein level by flow cytometry. In agreement with the RNA-Seq results, CNS-infiltrating *Gata3*-sufficient *Cd45.1/Cd45.2* CD4^+^ T effector cells expressed Egr2, while the late *Gata3*-knockout Cre-ERT2-*Gata3* ^fl/fl^ CD4^+^ effector cells expressed less Egr2 (**Figures 7A, B**). Bhlhe40 expression followed a similar pattern, with CNS-infiltrating *Cd45.1/Cd45.2* CD4 effector cells expressing higher levels of Bhlhe40 than the late *Gata3*-knockout CD4 effector cells (**Figures 7C, D**). Interestingly, in agreement with our prior observations that GATA3 expression is not restricted to RORγt^+^T-bet^+^ ‘pathogenic’ Th17 cells, and that the percentage of IFNγ^+^IL-17A^+^ Th17 and IFNγ^-^IL-17A^+^ Th17 cells were unaffected in late *Gata3*-knockout effector cells, the expression of Egr2 and Bhlhe40 were not restricted to RORγt^+^ cells. Instead, both RORγt^+^ Th17 and RORγt^-^ CD4 effector cells were able to express Egr2, Bhlhe40, and GM-CSF, suggesting that the GATA3-dependent expression of Egr2 and Bhlhe40 is not Th17 cell specific. To determine if early GATA3 might also affect the expression of Egr2 and Bhlhe40 in differentiating T cells, we revisited our early tamoxifen-inducible Cre-ERT2-*Gata3* ^fl/fl^ d6 draining lymph node *Gata3* deletion model. In the d6 draining lymph node, *Gata3*-sufficient vehicle control Cre-ERT2-*Gata3* ^fl/fl^ CD4 effector cells, including both RORγt^+^ Th17 and RORγt^-^ T cells, expressed Egr2 (**Figures 7E, F**) and Bhlhe40 (**Figures 7G, H**). On the other hand, tamoxifen-treated *Gata3*-knockout Cre-ERT2-*Gata3* ^fl/fl^ CD4 effector cells were largely Egr2 and Bhlhe40 negative (**Figures 7E–H**).

**Figure 8:**
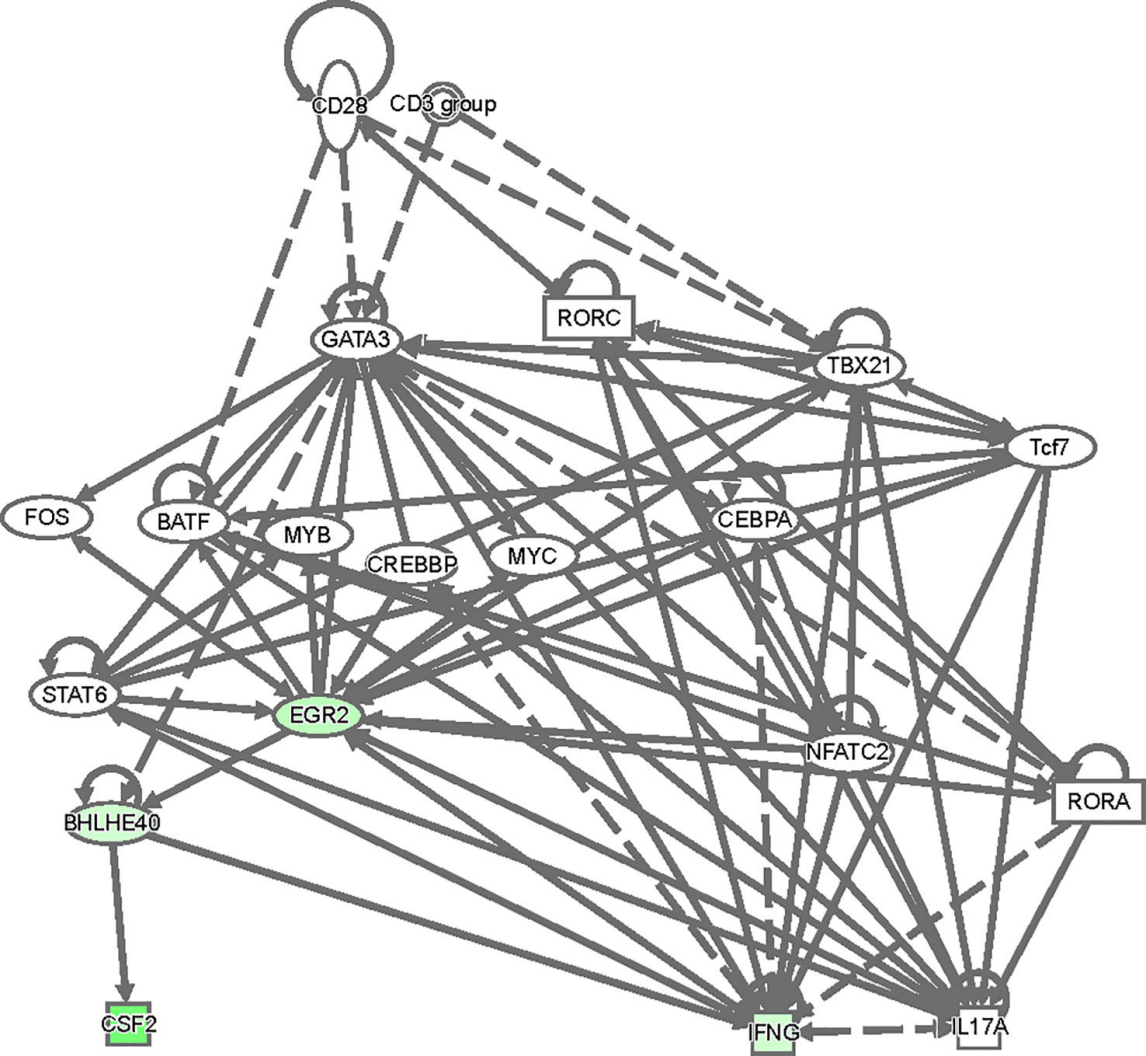
A model of regulatory network involving GATA3, Egr2, Bhlhe40 and Csf2. The top differentially expressed genes between CD4^+^CD44^hi^CD25^-^CD45.1^+^CD45.2^+^ C57BL/6 and CD45.1^-^CD45.2^+^ Cre-ERT2-*Gata3* ^fl/fl^ donor T effector cells in **Figure 6** were used in an ingenuity pathway analysis to visualize regulatory connections between GATA3, Egr2, Bhlhe40 and Csf2. Downregulated genes from the dataset were overlaid (green).

To gain insight as to how GATA3 might regulate *Bhlhe40* and thereby *Csf2* expression, we analyzed our RNA-Seq results in an Ingenuity Pathway Analysis (**Figure 8**). Based on this analysis, there are several ways in which TCR signaling-dependent GATA3 expression via Crebbp, Fos, Myc, Cebpa, and/or STAT6 might help induce *Egr2*. Egr2, or possibly GATA3 itself, may help to directly induce *Bhlhe40*, and Bhlhe40 in turn regulates *Csf2* expression resulting in GM-CSF-dependent encephalomyelitis in EAE.

## Discussion

GATA3 is the master transcription factor for Th2 cell differentiation and ILC2 development (53). It also plays an important role during T cell and ILC development at multiple stages (37, 54). In fact, GATA3 is expressed by all T cell and ILC subsets albeit at different levels (54, 55). We have previously reported that GATA3 regulates the development of NKp46^+^ ILC3s that express both RORγt and T-bet (35). Furthermore, it regulates the expression of IL-22 in ILC3s. In the present study, we found surprising new regulatory roles for GATA3 in regulating Th17 responses in autoimmune neuroinflammation. GATA3 expression is induced during *de novo* Th17 differentiation both *in vitro* and *in vivo*. Consistent with the previous finding that GATA3 regulates the development of T-bet/RORγt co-expressing ILC3s, it also regulates the differentiation of T-bet/RORγt co-expressing Th17 cells. Furthermore, continuous expression of GATA3 is required for GM-CSF expression in EAE.

In terms of the regulation of GATA3 expression, it is known that T cell receptor activation induces initial GATA3 expression *in vitro* under Th2 polarizing conditions, and GATA3 can help to enforce the Th2-program via a positive reinforcement loop involving autocrine IL-4 production (56, 57). Co-expression of RORγt and GATA3 may be explained by the induction of GATA3 within developing Th17 cells by IL-4 from a secondary cellular source. However, as our *in vitro* Th17 polarization conditions included an anti-IL-4 neutralizing antibody, TCR-driven expression of GATA3 in the absence of IL-4 would be the most likely explanation for the transient induction of GATA3 within developing Th17 cells.

We used three complimentary *Gata3* deletion models (Cre-ERT2-*Gata3* ^fl/fl^, *hCd2* ^Cre^ *Gata3* ^fl/fl^ and *Tbx21* ^Cre^ *Gata3* ^fl/fl^) to study the functions of an early GATA3 induction within nascent Th17 cells in EAE. These different models essentially yielded similar results: all three mouse strains were unable to develop notable EAE symptoms correlated with an inability to generate encephalitogenic T-bet^+^ Th17 cells within the draining lymph node or CNS following an immunization with MOG_35-55_. These results are noteworthy for several reasons. Firstly, despite the observation that RORγt and GATA3 are co-induced during Th17 differentiation, T-bet^-^RORγt^+^ Th17 cells were still able to develop with or without functional GATA3 protein; suggesting that Th17 cells do not intrinsically require GATA3 for their development, an observation that we have recently reported (38). Secondly, per these data, GATA3 is necessary for the development of encephalitogenic T-bet^+^ Th17 cells, presumably from T-bet^-^ Th17 cells. Thirdly, the deletion of *Gata3* did not result in an increase in IFNγ-production or T-bet^+^RORγt^-^ Th1 cells within the draining lymph nodes or CNS. These results were surprising as prior work *in vitro* has demonstrated that GATA3 actively represses Runx3 protein-regulated production of IFNγ within *in vitro* polarized Th2 cells (58) and T-bet and Runx protein are required for the development of pathogenic IFNγ-producing Th17 cells (59). Since *de novo* T-bet^+^ Th17 cells failed to develop in our *Gata3*-conditional knockout models, determining which genes are responsible for the observed phenotypes proved to be technically challenging and remains an open question. It is likely that GATA3 regulates the balance between RORγt and T-bet during the differentiation of T-bet^+^ Th17 cells as it does during the development of NKp46^+^ ILC3s.

Once GATA3 has been induced and subsequently downregulated, mature Th17 cells express low levels of GATA3. However, low levels of GATA3 expression are still required for eliciting EAE symptoms. In contrast to the effects of an early *Gata3* deletion on T cell priming, a late post-developmental deletion of *Gata3* did not affect the relative proportions of IFNγ^+^IL-17A^+^ or T-bet^+^RORγt^+^ ‘pathogenic’ Th17 cells in the CNS or periphery, but still prevented the development of encephalomyelitis symptoms. The presence of T-bet^+^RORγt^+^ ‘pathogenic’ Th17 cells allowed us to study gene regulation mediated by GATA3. Strikingly, this late *Gata3* deletion resulted in a defect in the production of GM-CSF, which has regarded as a pro-encephalomyelitic cytokine that is secreted by encephalitic Th17 and Th1 cells in the CNS (22–24). This effect is cell intrinsic as demonstrated by mixed congenic transfer EAE experiments. Transcriptomic analyses of *Gata3*-sufficient and *Gata3*-deficient (late *Gata3* deletion by tamoxifen) CNS-infiltrating CD4 effector cells from our mixed congenic transfer EAE model revealed stark reductions in the expression of *Bhlhe40* and *Egr2*, and enhanced expression of *Vhl* within late-*Gata3*-deficient CD4 effector cells. These results are noteworthy as in EAE experiments, CNS-infiltrating *Bhlhe40^-/-^* CD4 T cells are virtually unable to produce GM-CSF; Lin and colleagues have demonstrated that Bhlhe40 can directly regulate *Csf2* expression (31, 52). Like GATA3 (56, 57), *Bhlhe40* has been reported to be induced in response to TCR stimulation (31, 32); and Bhlhe40 and GM-CSF reporter mice have demonstrated that both are strongly expressed by CNS-infiltrating T cells in comparison to CD4 effector cells in the periphery (48, 52). These results suggest that TCR-dependent maintenance of GATA3 expression may help to regulate *Bhlhe40* and *Csf2* expression within the CNS. In addition, Vhl is an important regulator of the HIF1a hypoxic-response pathway in T cells (49) and has been implicated as a potential upstream regulator of *Bhlhe40* (*Stra13*) in human RCC4 cells (50). Lastly, Miao and colleagues have demonstrated that Egr2 can bind to the *Bhlhe40* locus within CD4 T cells in ChIP experiments (51). Since both Bhlhe40 and Egr2 are also regulated by early TCR signaling, it is likely that GATA3 is required for the maintenance of Bhlhe40 and Egr2 expression within the CNS which leads to GM-CSF production by encephalitic Th17 cells. While the results presented here highlight a novel role for GATA3 in regulating GM-CSF production, the results are limited in that it is unclear if the effects of GATA3 on GM-CSF are direct, indirect via regulation of Egr2 and Bhlhe40, or a mixture thereof. Additional studies will be needed to determine the exact regulatory mechanisms though which GATA3 affects T cell intrinsic GM-CSF production.

Altogether, our data demonstrate that dynamic GATA3 expression during Th17 cell differentiation is required for Th17-mediated encephalomyelitis in EAE. An early deletion of GATA3 during Th17 cell differentiation blocked the development of ‘pathogenic’ T-bet^+^RORγt^+^ Th17 cells, however, a late deletion of GATA3 at the established T effector stage allowed the presence of T-bet^+^RORγt^+^ Th17 cells. Nevertheless, GATA3 is still critically required for encephalomyelitis, which is associated with a reduction in the expression of GM-CSF and its regulators. Thus, our study highlights a novel role for GATA3 in promoting the pathogenicity of T-bet^+^ Th17 cells in EAE, via putative regulation of Egr2, Bhlhe40, and GM-CSF expression.

## Data availability statement

The datasets presented in this study can be found in online repositories. The names of the repository/repositories and accession number(s) can be found below: GSE227394 (GEO), https://www.ncbi.nlm.nih.gov/geo/query/acc.cgi?acc=GSE227394.

## Ethics statement

The animal study was reviewed and approved by National Institute of Allergy and Infectious Diseases (NIAID) Animal Care and Use Committee.

## Author contributions

JZ conceived the project. MB performed most of the experiments. RG and XZ performed some *in vitro* and *in vivo* experiments. XC contributed to the RNA-Seq experiments. GH and MB performed bioinformatic analysis. VL and KZ made intellectual contributions and edited the manuscript. MB and JZ wrote the manuscript. JZ supervised the project. All authors contributed to the article and approved the submitted version.
